# Isolated Splenic Lymphangiomas Presenting in an Infant with Isolated Anaemia

**DOI:** 10.1155/2020/8919424

**Published:** 2020-04-13

**Authors:** Ameer Kakaje, Raghad Mujahed, Othman Hamdan

**Affiliations:** ^1^Damascus University, Damascus, Syria; ^2^Department of Haematology, Children's University Hospital, Damascus University, Damascus, Syria

## Abstract

Lymphangiomas are benign tumours of lymphatic vessels which are not linked with the normal lymphatic vessels. Their symptoms usually depend on their location and size as they can compress or block adjacent organs. We present a 9-month-old girl with isolated cavernous lymphangiomas of the spleen, a rare variable of lymphangiomas, with no symptoms. She had an isolated anaemia which could only be explained by the hypersplenism caused by the lymphangiomas. Such a presentation has been very rarely reported in the literature and not mainly in children. Furthermore, this case also illustrates imaging of hypersplenism related to lymphangiomas.

## 1. Introduction

Lymphangiomas are benign tumours of the lymphatic vessels. They are typically at the neck and axilla in 95% of the cases [1]. They mainly occur in children, but are rarely located in the spleen. They frequently affect many organs simultaneously in a context of a syndrome called “lymphangiomatosis”. Patients hardly ever have isolated lymphangiomas of the spleen as only few cases were reported in the literature [1, 2]. Lymphangiomas of the spleen are often asymptomatic and diagnosed incidentally by imaging [2]. We only found one case in the literature that presented with an isolated anaemia, and it was in an adult [3]. This made this case quite unique as it was in an infant with no other explanation for the anaemia, and it provided imaging of the lymphangiomas of the spleen that caused this phenomenon.

## 2. Case Report

**Figure 1:**
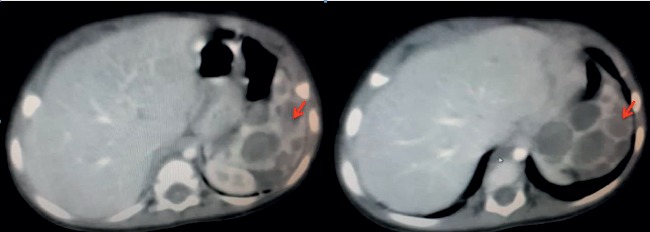
CT scan of the abdomen that shows the lymphangioma cysts (red arrow) of the spleen.

**Figure 2:**
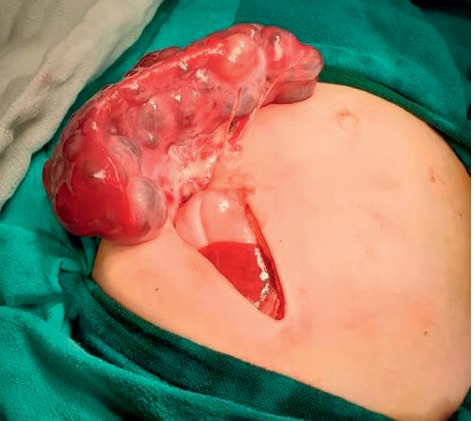
The spleen with the lymphangioma cysts that caused the hypersplenism.

A 9-month-old Arab girl came to the clinic for a regular examination. She was breastfed, and her diet was adequate for her age. She did not have a remarkable medical or family history. Her delivery was normal and uncomplicated. She did not report a loss of appetite or weight loss. On examination, the child was playful and afebrile with no dyspnoea, fever, or cough. However, the spleen was palpable by 4 cm under the left costal line. Her labs showed a microcytic anaemia (Hb = 9.2 g/dl and MCV = 64 fl/cell; normal values: Hb > 11.3 g/dl and MCV > 70 for her age). Iron studies were normal. Direct and indirect Coombs tests were negative, and electrophoresis was normal. Her white blood cell count and platelet count were normal according to her age. PT, PTT, albumin, and liver function tests were all normal. Hepatitis B and C were also excluded by serological tests. Ultrasonography showed an enlarged spleen, measuring 9.2 cm in the larger diameter with several cysts in the parenchyma which was heterogeneous. The hypoechoic cysts' walls were regular with mild calcifications and had septa and unclear fluid. Hepatic and portal veins were all normal. Chest X-ray was normal. An abdominal computed tomography (CT) scan (Figure 1) showed an enlarged, heterogeneous spleen which measured 5.5 × 9 cm. The mass had solid and cystic components with different diameters. The cysts' walls were regular with mild calcifications. The lesions were only found in the spleen without other lymphatic vessel lesions. No organomegaly, adenopathy, skin lesions, or any other abnormalities were found. Differential diagnosis was lymphangiomas, hemangiomas, epidermoid cysts, mesothelial cysts, and parasitic cysts and needed confirmation with pathology. No other lesions were observed in the abdomen, pelvis, head, neck, limbs, or chest regions. Splenectomy was indicated; in surgery, a left subcostal (Kocher) incision was made, revealing the enlarged spleen with multiple cysts (Figure 2). A total surgical splenectomy was performed, and the pathology report confirmed the diagnosis of cavernous lymphangiomas. The child was discharged four days after the surgery, and regular follow-ups showed the improvement of the anaemia which was back to normal after three months.

## 3. Discussion

Lymphangiomas are congenital malformations that affect lymphatic vessels, causing them to dilate. These dilations are not connected with the normal lymphatic system, and they contain proteinaceous fluid [4]. Lymphangiomas are divided into circumscriptum and cavernous, with the latter being deeper and rising in the loose connective tissue. Overall, approximately 90% of lymphangiomas are found in children and usually diagnosed before two years of age. They are also more common in females [2, 5].

Lymphangiomatosis syndrome should be excluded, and thus a full-body scan is needed to exclude any other affected organ [2]. We performed a full-body examination and thoracoabdominal CT scan to exclude other lesions. Despite the size of the tumour, our patient did not have dyspnoea, or any other symptom. The patient only had an abdominal distension and anaemia. Bone aspiration was normal, and the patient did not have a haemorrhagic syndrome, and therefore the anaemia could only be explained by hypersplenism, caused by lymphangiomas. This has been previously reported in an adult patient who only had anaemia and a sporadic lymphangioma in the spleen [3], but our case is the first in a child.

Spleen with lymphangiomas can be of normal size or enlarged, and the radiological findings are not specific; lymphangiomas are usually well defined and found as hypoechoic or anechoic cysts with septa or calcifications [1, 2]. Having calcifications in the peripheral wall on imaging is indicative of lymphangiomas [1]. They are also seen on CT scans as thin-walled cysts with low attenuation or contrast enhancement. Lymphangiomas are usually found in the subcapsular region but may be found in the parenchyma when the tumours are large [1, 2]. Splenic cysts are presented on the CT scan as spherical and well-defined lesions that have an attenuation close to water with thin walls that have no enhancement [6]. However, many focal lesions may appear as cysts in the spleen, and thus the differential diagnosis can be wide; epithelial splenic cysts comprise 25% of true cysts, and they are mainly in children and young adults. However, they are mostly solitary [6]. Parasite cysts from a flatworm infection that forms a hydatid cyst are common in Syria. However, they are extremely rare in the spleen even in endemic areas [6]. Calcifications can be also seen with close features of other cysts [6]. Benign tumours such as cavernous hemangiomas can also be confused with other cysts as they can present on a CT scan as cystic lesions with iso- or hypodense areas and sometimes with peripheral calcifications and centripetal enhancement on sequential CT images [6].

Splenic lymphangiomas are mostly benign, and malignance transformation can rarely occur [1]. Total splenectomy is the treatment of choice for isolated splenic lymphangiomas as other options are not as effective [1, 5] so that severe complications will be avoided such as splenic rupture and haemorrhage [2]. Most symptoms are nonspecific and depend on the size as the enlarged spleen can compress the adjacent tissue such as the renal tissue or the stomach and diaphragm. They can also present with acute abdomen when they are complicated such as rupture, or with consumptive coagulopathy and bleeding [2, 7]. The association between hypersplenism and lymphangioma has also been reported [7]. Moreover, complications such as consumptive coagulopathy and hypersplenism are positively correlated with the size or extend of lymphangiomas of the spleen [8]. However, they did not illustrate the imaging of splenic lymphangiomas in cases of hypersplenism such as CT scans. Moreover, the prevalence of hypersplenism in lymphangiomas and their size havenot been studied.

In conclusion, the 9-month-old female patient had isolated lymphangiomas of the spleen with no other lesion. She was asymptomatic and had an isolated anaemia which has been rarely reported as a complication of splenic lymphangiomas which makes this case quite unique. This case also provided the imaging of such a presentation.
